# The emergence of DNA in the RNA world: an *in silico* simulation study of genetic takeover

**DOI:** 10.1186/s12862-015-0548-1

**Published:** 2015-12-07

**Authors:** Wentao Ma, Chunwu Yu, Wentao Zhang, Sanmao Wu, Yu Feng

**Affiliations:** College of Life Sciences, Wuhan University, Wuhan, 430072 P.R.China; College of Computer Sciences, Wuhan University, Wuhan, 430072 P.R.China

**Keywords:** Origin of life, Molecular evolution, Computer modeling

## Abstract

**Background:**

It is now popularly accepted that there was an “RNA world” in early evolution of life. This idea has a direct consequence that later on there should have been a takeover of genetic material – RNA by DNA. However, since genetic material carries genetic information, the “source code” of all living activities, it is actually reasonable to question the plausibility of such a “revolutionary” transition. Due to our inability to model relevant “primitive living systems” in reality, it is as yet impossible to explore the plausibility and mechanisms of the “genetic takeover” by experiments.

**Results:**

Here we investigated this issue by computer simulation using a Monte-Carlo method. It shows that an RNA-by-DNA genetic takeover may be triggered by the emergence of a nucleotide reductase ribozyme with a moderate activity in a pure RNA system. The transition is unstable and limited in scale (i.e., cannot spread in the population), but can get strengthened and globalized if certain parameters are changed against RNA (i.e., in favor of DNA). In relation to the subsequent evolution, an advanced system with a larger genome, which uses DNA as genetic material and RNA as functional material, is modeled – the system cannot sustain if the nucleotide reductase ribozyme is “turned off” (thus, DNA cannot be synthesized). Moreover, the advanced system cannot sustain if only DNA’s stability, template suitability or replication fidelity (any of the three) is turned down to the level of RNA’s.

**Conclusions:**

Genetic takeover should be plausible. In the RNA world, such a takeover may have been triggered by the emergence of some ribozyme favoring the formation of deoxynucleotides. The transition may initially have been “weak”, but could have been reinforced by environmental changes unfavorable to RNA (such as temperature or pH rise), and would have ultimately become irreversible accompanying the genome’s enlargement. Several virtues of DNA (versus RNA) – higher stability against hydrolysis, greater suitability as template and higher fidelity in replication, should have, each in its own way, all been significant for the genetic takeover in evolution. This study enhances our understandings of the relationship between information and material in the living world.

**Electronic supplementary material:**

The online version of this article (doi:10.1186/s12862-015-0548-1) contains supplementary material, which is available to authorized users.

## Background

In modern organisms, DNA is the major genetic material and protein is the major functional material, both seeming indispensible. Thus, there is a dilemma for the evolution of life: “Which came first, DNA or proteins?” The RNA world hypothesis provided a possible solution to this problem: in some early stage of life, there is neither DNA nor protein but only RNA, acting as both genetic material and functional material [1]. The RNA world hypothesis has gain more and more supporting evidence, and has become the most popular idea in the field of the origin of life [2–5]. Indeed, in reality, peptides are likely to have existed in the RNA world, considering that they may have been easy to synthesize prebiotically and may have been able to aid RNA’s function by forming complexes with RNA [6, 7]. However, these peptides, synthesized abiotically, cannot reappear in the next generation anyway (unlike those encoded proteins emerging later). Thus, the stage was still an “RNA world”, considering chemical existence is not sufficient to “justify” the existence of a substance in a living world (more or less, the peptides can be seen as merely environmental factors, like metal ions, for example). Recently, a significant study suggested that precursors of RNA, proteins and lipids may have derived by common chemistry [8] – while the result was inspiring for researchers working in the field of the origin of life, a clear notion concerning the difference between chemical existence and biotic existence, like mentioned here, becomes particularly important.

As a clear consequence of the scenario concerning the RNA world, DNA and proteins (encoded) should have appeared afterwards. For the emergence of DNA, there would be a problem of “genetic takeover”; for the emergence of proteins, there would be a problem of “functional takeover”. Indeed, “after RNA, which came first, DNA or proteins?” is still a question (e.g., [9] and [10]). While problems concerning such critical transitions remain difficult to tackle experimentally, they begin to appear within the reach of computer simulation researches.

The present computer simulation study focuses on the genetic takeover and follows the idea of DNA emerging before proteins. Perhaps the authors’ own opinions about the emerging order of DNA and proteins are not important here, and the choice could simply be technical: the genetic takeover is simpler in principle than the functional takeover given that RNA and DNA share the same mechanism in their synthesis, i.e., by base pairing (unlike that in proteins’ synthesis). In particular, we have conducted a series of computer simulation studies concerning the scenario of early development of the RNA world, including the emergence of several important ribozymes [11–13], the cooperation of these ribozymes [14], and the emergence of an RNA “chromosome” (with linked genes coding for the ribozymes) [15]. In fact, it is now feasible, by a slight extension of the approach along this line, to investigate “the emergence of a DNA chromosome” – ultimately, the transition from an RNA world to a DNA/RNA world.

Indeed, for this transition to occur, additional functions concerning new kinds of template-directed copying should be required, i.e., the cross copying between DNA and RNA as well as DNA’s replication. However, these new kinds of copying may have been initially catalyzed by that “old” ribozyme, i.e., the ribozyme that catalyzed RNA’s own replication (the “RNA replicase” – “Rep” for short) [2, 16], given the similar chemistry behind all these reactions [10, 17]. Other than this, it appears that only a nucleotide reductase ribozyme (“Nrr”), which synthesizes deoxynucleotides from nucleotides, is needed [10, 17]. Therefore, here we could start our simulation study simply with the introduction of such a ribozyme. Our concerns are: could the so-called genetic takeover take place in our model system? If it could, what about its mechanisms? (These mechanisms may be reflected by the influence of some key parameters on the dynamics of the model system).

## Results

Practically, the present model was derived directly from the one used in our recent study [15], in which the system was distributed with protocells containing an RNA chromosome and several ribozymes it codes, i.e., a Rep, a nucleotide synthetase ribozyme (“Nsr”), a nucleotide precursor synthetase ribozyme (“Npsr”), and an amphiphile (membrane component) synthetase ribozyme (“Asr”). Here the simulation study started with the introduction of a fifth ribozyme, an Nrr. See “Methods” for a detailed description of the model.

### The influence of a moderate nucleotide reductase ribozyme

**Fig. 1:**
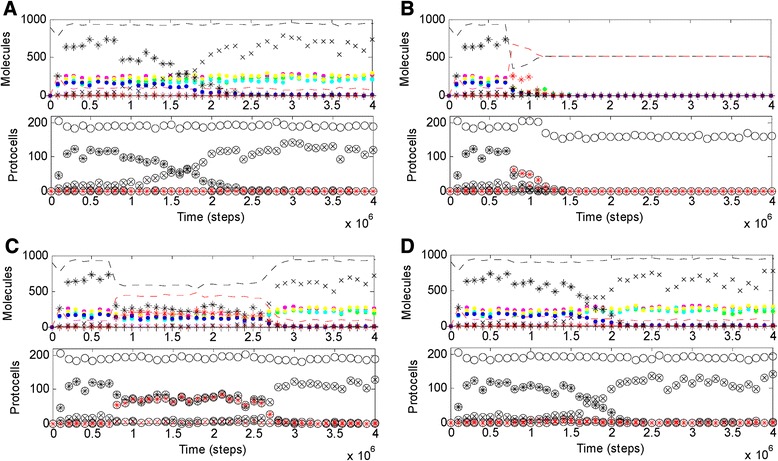
The influence of Nrr with different active rates on the system of the RNA-based protocells. (**a**) The Nrr’s active rate (*P*_*NRR*_) is kept 0 throughout the simulation. Based on the case shown in *A*, at step 0.7 × 10^6^, *P*_*NRR*_ is changed from 0 to (**b**) 0.5; (**c**) 0.02; (**d**) 0.001. For each subfigure, the top panel shows the dynamics at the molecular level, and the bottom panel the cellular level. Stars represent the chromosome (RNA in black and DNA in *red*) containing all the five genes, while x-shapes represent the chromosome (RNA in *black* and DNA in *red*) containing only four genes (lacking the Nrr). The black dashed line denotes the total materials concerning RNA, including RNA, nucleotides, nucleotide precursors and nucleotide precursors’ precursors (all counted as quotients in measurement of nucleotides; and to be drawn in this same figure, the “materials” are here represented in a 1/200 scale); and in a similar way the red dashed line denotes the total materials concerning DNA. Dots represent the ribozymes (Rep in *magenta*, Nsr in *green*, Npsr in *cyan*, Asr in *yellow*, and Nrr in *blue*). Empty circles represent total protocells, and the circles with a symbol in represent the protocells containing the chromosome represented by that symbol (e.g., the circles with a black star in represent the protocells containing the five-gene RNA chromosome). Note that the symbols in the following dynamic figures are interpreted the same way

The RNA chromosome now has five genes. However, to trace the effect of the Nrr gene, it is first assumed that the ribozyme Nrr cannot work (i.e., *P*_*NRR*_ = 0, see Tables 1 and 2 for the descriptions of parameters). After the initial inoculation (see “Methods”), the chromosome and the ribozymes, as well as the protocells containing them, spread (proliferate) in the system (Fig. 1a), which is consistent with our previous study [15]. However, some time later (at about step 0.9 × 10^6^ in the case shown here), the chromosome (black stars) begins to decrease while the chromosome lacking the Nrr gene (black x-shapes) begins to increase, which, taken together, mean the “losing” of the Nrr gene. A similar trend can be observed at the cellular level (see the bottom panel). Indeed, the ribozyme Nrr (blue points) also decreases. Finally, the Nrr gene goes into extinction (actually, it is altered in sequence rather than deleted – the chromosome’s length remains unchanged – refer to Additional file 1: Figure S1A). This “phenomenon” is well consistent with our understandings that a non-functional sequence would not be conserved (because of random mutations).

**Table 1:** Parameters used in the model (part 1^a^)

Probabilities	Descriptions	Values^b^
*ᅟP*_*DND*_	Deoxynucleotide decaying into its precursor	5 × 10^−4^
*ᅟP*_*DNDE*_	Deoxynucleotide decaying into its precursor at DNA’s chain end	1 × 10^−5^
*ᅟP*_*DNF*_	Deoxynucleotide forming from its precursor	5 × 10^−4^
*ᅟP*_*DNO*_	Deoxynucleotide being oxidized^c^	1 × 10^−4^
*ᅟP*_*DNPD*_	Deoxynucleotide precursor decaying into its precursor	5 × 10^−4^
*ᅟP*_*DNPF*_	Deoxynucleotide precursor forming from its precursor	5 × 10^−4^
*ᅟP*_*DNPO*_	Deoxynucleotide precursor being oxidized^c^	1 × 10^−4^
*ᅟP*_*DNPPO*_	Deoxynucleotide precursor’s precursor being oxidized^c^	1 × 10^−4^
*ᅟP*_*FPDD*_	False base-pairing when DNA attracting deoxynucleotides/DNA	0.001
*ᅟP*_*FPDR*_	False base-pairing when DNA attracting nucleotides/RNA	0.02
*ᅟP*_*FPRD*_	False base-pairing when RNA attracting deoxynucleotides/DNA	0.01
*ᅟP*_*NPPR*_	Nucleotide precursor’s precursor being reduced^c^	1 × 10^−4^
*ᅟP*_*NPR*_	Nucleotide precursor being reduced^c^	1 × 10^−4^
*ᅟP*_*NR*_	Nucleotide being reduced^c^ (not catalyzed)	1 × 10^−4^
*ᅟP*_*NRR*_	Nucleotide being reduced^c^ (catalyzed by Nrr)	---
Others	Descriptions	Values^b^
*ᅟF*_*BBD*_	Factor for phosphodiester-bond-breaking in DNA (vs RNA)	0.01
*ᅟF*_*SFD*_	Factor for single DNA chain folding	1
*ᅟF*_*SFR*_	Factor for single RNA chain folding	2

^a^This part contains parameters newly introduced here. See Table 2 for the others, which were also used in the previous model [15]

^b^These parameter values, as well as those shown in Table 2, are for the cases shown in Figs. 1, 2, 3 (about the adjustment of some key parameters during simulation in these cases, see the figures’ legend for details). There are only a few differences for the cases shown in Fig. 4, which have been elucidated in the text. As explained previously [14, 15], such values are adopted according to logic, knowledge (see also “Methods” of this paper) and experience, and though they are listed here with an “accurate appearance”, the dynamics of the model system is generally not sensitive to their moderate changes

^c^Here the oxidation and reduction mean the mutual transformation between deoxynucleotides and nucleotides, between their precursors, or between their precursors’ precursors

**Table 2:** Parameters used in the model (part 2^a^)

Probabilities	Descriptions	Values
ᅟ*P*_*AD*_	Amphiphile decaying into its precursor (out of membrane)	5 × 10^−4^
ᅟ*P*_*ADM*_	Amphiphile decaying into its precursor within membrane	5 × 10^−5^
ᅟ*P*_*AF*_	Amphiphile forming from its precursor (not catalyzed)	5 × 10^−4^
ᅟ*P*_*AFR*_	Amphiphile forming from its precursor (catalyzed by Asr)	0.5
*ᅟP*_*AJM*_	Amphiphile joining membrane	0.9
*ᅟP*_*ALM*_	Amphiphile leaving membrane	5 × 10^−5^
*ᅟP*_*APP*_	Amphiphile precursor permeating membrane	0.05
*ᅟP*_*AT*_	Nucleic acid template attracting momomers/oligomers by base-pairing	0.2
*ᅟP*_*BBR*_	Phosphodiester bond breaking in an RNA chain	2 × 10^−6^
ᅟ*P*_*CB*_	Protocell breaking	1 × 10^−5^
*ᅟP*_*CD*_	Protocell dividing	0.005
*ᅟP*_*CF*_	Protocell fusing	5 × 10^−4^
*ᅟP*_*CTT*_	A circular nucleic acid chain turning to a template	0.9
*ᅟP*_*EL*_	End-to-end ligation of a nucleic acid chain (cyclization)	1 × 10^−7^
*ᅟP*_*FLR*_	Ligating with false base-pairing on template (by Rep)	0.1
*ᅟP*_*FPRR*_	False base-pairing when RNA attracting nucleotides/RNA	0.01
*ᅟP*_*LTT*_	A linear nucleic acid chain turning to a template	0.01
*ᅟP*_*MC*_	Movement of a protocell	0.05
*ᅟP*_*MF*_	Membrane forming	0.1
*ᅟP*_*MV*_	Movement of a (deoxy/)nucleotide, amphiphile or 2their precursors	0.5
*ᅟP*_*ND*_	Nucleotide decaying into its precursor	5 × 10^−4^
*ᅟP*_*NDE*_	Nucleotide decaying into its precursor at RNA’s chain end	1 × 10^−5^
*ᅟP*_*NF*_	Nucleotide forming from its precursor (not catalyzed)	5 × 10^−4^
*ᅟP*_*NFR*_	Nucleotide forming from its precursor (catalyzed by Nsr)	0.5
*ᅟP*_*NPD*_	Nucleotide precursor decaying into its precursor	5 × 10^−4^
*ᅟP*_*NPF*_	Nucleotide precursor forming from its precursor (not catalyzed)	5 × 10^−4^
*ᅟP*_*NPFR*_	Nucleotide precursor forming from its precursor (catalyzed by Npsr)	0.5
*ᅟP*_*NPP*_	(Deoxy/)nucleotide precursor permeating membrane	0.01
*ᅟP*_*NPPP*_	(Deoxy/)nucleotide precursor’s precursor permeating membrane	0.2
*ᅟP*_*RB*_	Rep binding onto a nucleic acid template	0.9
*ᅟP*_*RD*_	Rep dropping from a nucleic acid template	0.9
*ᅟP*_*RL*_	Random ligation of RNA with RNA or that of DNA with DNA	1 × 10^−7^
*ᅟP*_*SP*_	Separation of a base pair	0.5
*ᅟP*_*TL*_	Template-directed ligation (not catalyzed)	5 × 10^−4^
*ᅟP*_*TLR*_	Template-directed ligation (catalyzed by Rep)	0.5
Others	Descriptions	Values
*ᅟF*_*DE*_	Factor for the effect of Donnan’s equilibrium	5
*ᅟF*_*DO*_	Factor for the degradation/decay of molecules out of protocells	100
*ᅟF*_*IB*_	Factor for intermediate RNA breaking (at sites between genes)	1000
*ᅟF*_*OP*_	Factor for the effect of osmotic pressure	5
ᅟ*L*_*AM*_	Lower limit of amphiphiles to form protocell membrane	600
*ᅟN*	The system surface is defined as an N × N grid.	60
*ᅟT*_*APB*_	Total amphiphile precursors introduced in the beginning	1.2 × 10^5^
*ᅟT*_*NPPB*_	Total nucleotide precursors’ precursors introduced in the beginning	1.8 × 10^5^

^a^This part of parameter list is derived directly from the parameter list of the previous model [15]. *P*_*BBR*_ was originally named *P*_*BB*_, *P*_*FPRR*_ originally *P*_*FP*_, *P*_*CTT*_ originally *P*_*CRTT*_, and *P*_*LTT*_ originally *P*_*LRTT*_. To suit the aim of the present model, into which DNA is introduced,the roles of *P*_*AT*_, *P*_*CTT*_, *P*_*EL*_, *P*_*LTT*_, *P*_*MV*_, *P*_*NPP*_, *P*_*NPPP*_, and *P*_*RL*_ have been modified (see “Descriptions”), and the “working values” of *P*_*AFR*_, *P*_*CD*_, *P*_*FLR*_, *P*_*NDE*_, *P*_*NFR*_, *P*_*NPFR*_, *P*_*TLR*_, *F*_*DO*_ and *F*_*IB*_ are adjusted to some extent. The values of *N*, *T*_*NPPB*_ and *T*_*APB*_ are enlarged to increase the scale of the model system, and thus the dynamics of the present model system is more robust against casual events and bears a better statistical property

In the model, DNA is assumed to bear several virtues relative to RNA (as genetic material) according to our knowledge. First, DNA is more stable against chain break [18] (here 100 times, i.e., *F*_*BBD*_ = 0.01). Second, DNA is easier to act as a template (i.e., less likely to self-fold [19]) (here twice, i.e., *F*_*SFR*_ = 2 while *F*_*SFD*_ = 1). Third, DNA is less error-prone in replication [20] (here 10 times, i.e., *P*_*FPRR*_ = 0.01 while *P*_*FPDD*_ = 0.001). Here, the consideration that the mutation rate for DNA replication should be lower than that of RNA replication is relevant to their template feature (unrelated to enzymes), and so the difference could not be very great [20]. Note that DNA or RNA may act as a copying template for each other; and the Rep is assumed to be able to aid all the four kinds of copying: RNA to RNA, RNA to DNA, DNA to DNA and DNA to RNA (the type of the product, RNA or DNA, depends on the foregoing substrate in the copying – see “Methods”). Then, our concern is: if the activity of Nrr is “turned on”, would a genetic takeover occur? Correlatively, given its function favoring the emergence of DNA, would the Nrr’s extinction be prevented?

The active rate of Nrr, *P*_*NRR*_ is changed from 0 to 0.5 (a rate equal to those of the other ribozymes in the model) at a step before the Nrr gene begin to fade away (step 0.7 × 10^6^ in the case shown in Fig. 1a). Somewhat unexpectedly, the system collapses directly after this “key step” (Fig. 1b). The number of the RNA chromosome (black stars) drops immediately while the DNA chromosome (red stars) rises. The RNA material as a whole (black dashed line) drops while the DNA material (red dashed line) rises. Significantly, the ribozymes (dots in different colors) decrease meanwhile. In other words, the Nrr could cause the rise of DNA, but would lead to the drop of RNA, which includes the indispensible ribozymes – thus resulting in the system’s collapse. When the system collapse, the RNA material and the DNA material take a trend towards the same level – this is a reasonable result because given no “biotic” effect is available, the balance is determined by those “abiotic” rates, the non-enzymatic “inter-transforming” rates between nucleotides and deoxynucleotides, between nucleotide precursors and deoxynucleotide precursors, and between nucleotide precursors’ precursors and deoxynucleotide precursors’ precursors, which are in this case set to be equal respectively (i.e., *P*_*NR*_ = *P*_*DNO*_; *P*_*NPR*_ = *P*_*DNPO*_; *P*_*NPPR*_ = *P*_*DNPPO*_, see Table 1).

**Fig. 2:**
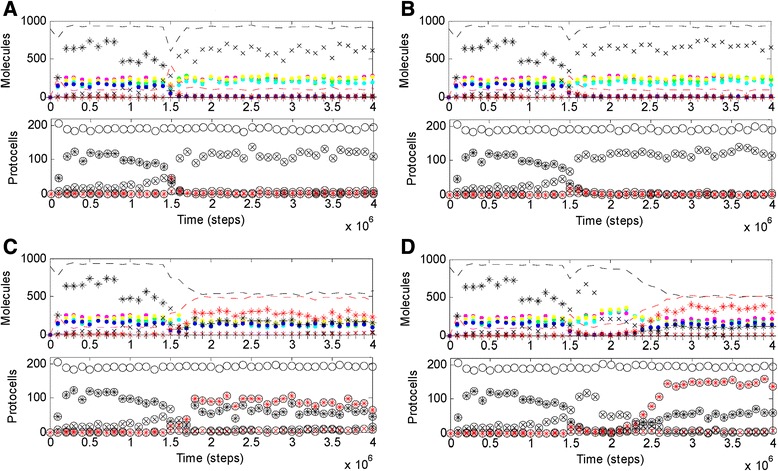
Several cases showing the dynamics of the model system when the Nrr’s effect is not “applied on a global scale”. Based on the case shown in Fig. 1a, at step 1.4 × 10^6^, *P*_*NRR*_ is changed from 0 to (**a**) 0.5; (**b**) 0.02. Based on the case shown in *B*, (**c**) at step 1.5 × 10^6^, *F*_*SFR*_ is changed from 2 to 10; (**d**) at step 1.8 × 10^6^, *P*_*BBR*_ is changed from 2 × 10^−6^ to 2 × 10^−5^. Note that at step 1.4 × 10^6^, due to random mutation, apparently only a portion of the RNA chromosome molecules maintains the “non-functional” Nrr gene (see the descending of black stars, which denote the five-gene chromosome, and the rising of black x-shapes, which denote the four-gene chromosome). So when the activity of the Nrr is turned on at this moment, the Nrr’s effect is only “applied on a limited scale”. The thorough genetic takeover shown in *D* is in fact originated from a single individual, because at step 1.8 × 10^6^ in this case there are actually only one protocell containing the five-gene chromosome, which bears the moderate Nrr gene

In reality, a deleterious gene would appear through mutation in an individual, and the result, at most, may be the elimination of the individual together with the gene in the population, rather than a system-wide collapse. Clearly, by the way of dynamic simulation as shown here, it would be hard to judge such gene elimination concerning single mutants, because if an individual disappears, the disappearance may also be caused by some random event. However, we may do a “conservative simulation” to show the phenomenon. For the case shown in Fig. 1a, if *P*_*NRR*_ is changed from 0 to 0.5 at step 1.4 × 10^6^ – when only a portion of the chromosome molecules still maintain the sequence of the Nrr gene, we can see the gene-elimination phenomenon (instead of the system-wide collapse), in which the Nrr’s extinction is accelerated (Fig. 2a, compared with Fig. 1a). The logic to say this is a conservative simulation is that the deleterious gene would be eliminated from the system even if a number of individuals contain it, let alone in the case that it appears within only one individual through mutation. On the other side of the coin, we get to know that the reason for the system-wide collapse shown in Fig. 1b is that the “deleterious gene” is here “applied suddenly” on a globe scale – i.e., in all individuals (at step 0.7 × 10^6^), and no individual escapes from the elimination.

Does the system collapse or the gene elimination result from the high active rate of Nrr (*P*_*NRR*_ = 0.5)? Obviously, Nrr is not a pure “bad” gene given that it favors the synthesis of DNA, a better genetic material. So then, lower active rates were tested here. Indeed, when *P*_*NRR*_ is changed from 0 to 0.02 (instead of 0.5) at the “key” step (step 0.7 × 10^6^), the result turns out with an appearance somewhat closed to the genetic takeover we expected (Fig. 1c). A “platform” sustains for quite a long time, in which the five-gene DNA chromosome (red stars) co-exists with the five-gene RNA chromosome (black stars) (see also Additional file 1: Figure S1B). However, then (at about 2.6 × 10^6^ step) these chromosomes begin to descend, while the four-gene RNA chromosome (lacking Nrr; black x-shapes) begins to rise (like the situation in the second half of Fig. 1a). This means that the transition is still unstable, and might turn back towards the pure RNA system. This is no longer a problem related to the Nrr’s active rate – with a greater rate the gene would appear too deleterious, whereas with a lower rate the gene would be too “weak” with regard to its benefit side (Fig. 1d shows that an Nrr with only a rate of 0.001 has nearly no influence on the dynamics of the system – compared with Fig. 1a; see Additional file 1: Figure S2 for more detailed results concerning the influence of the Nrr’s active rate).

### Towards a thorough genetic takeover

**Fig. 3:**
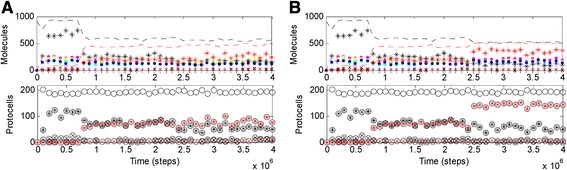
The stabilization of the DNA/RNA platform that is brought about by the moderate Nrr. Based on the case shown in Fig. 1c, at step 2.4 × 10^6^, (**a**) *F*_*SFR*_ is changed from 2 to 10 (the template activity of RNA is turned down); (**b**) *P*_*BBR*_ is changed from 2 × 10^−6^ to 2 × 10^−5^ (the rate of RNA’s chain break is turned up)

It seems that the unstability of the transition stems from the “counterattack” of the four-gene RNA-based individuals, which though lacking a better genetic material, did not have to endure a shortage of building blocks for ribozymes. Indeed, when the template activity of RNA is turned down deliberately (*F*_*SFR*_ is changed from 2 to 10 at step 2.4 × 10^6^), which means that an RNA chromosome is further disfavored, the four-gene RNA chromosome no longer rises and the DNA/RNA platform is saved (Fig. 3a). Moreover, the five-gene DNA chromosome (red stars) ascends to a level higher than the five-gene RNA chromosome (black stars) (see also Additional file 1: Figure S1C). Indeed, this represents a veritable genetic takeover – the DNA chromosome could already sustain itself along by replication (Additional file 1: Figure S3A). However, the RNA chromosome would not disappear – it would be mainly copied from the DNA chromosome (Additional file 1: Figure S3A); thus, rather than a chromosome, it is, to a greater extent, a transcript of the DNA chromosome (and certain chain-breakings of the transcript may give rise to the ribozymes, see [15] for details).

Imaginably, just due to the competition from the individuals around that lacked Nrr, in reality, a mutant with the moderate Nrr might hardly bring about a “system-wide” platform. As a relevant case of “conservative simulation”, the global effect cannot appear even if more than half of the individuals in the system get the function simultaneously (Fig. 2b, i.e., *P*_*NRR*_ is changed from 0 to 0.02 at step 1.4 × 10^6^, when many of the chromosomes/protocells still maintain the Nrr sequence – the black star is higher than the black x-shape in the upper panel, and the circle with a black star is higher than the circle with a black x-shape in the lower panel). Nonetheless, “local sparks” of the moderate Nrr should still be significant for potential genetic takeover – indeed, their effect can spread to a global scale if RNA’s template activity is turned down (Fig. 2c, *F*_*SFR*_ is changed from 2 to 10 at step 1.5 × 10^6^). It seems that what they are waiting is simply a chance, when the DNA chromosome’s advantage over the RNA chromosome is enhanced.

In this regard, though it seems difficult to tell what kinds of event in reality may attenuate RNA’s template activity (corresponding to the adjustment of *F*_*SFR*_ analyzed above), we can easily imagine the environmental changes that disfavor the fragile RNA chromosome, e.g., the rise of temperature or pH value. To simulate such a change, the chain-breaking rate of RNA is turned up, i.e., *P*_*BBR*_ is changed from 2 × 10^−6^ to 2 × 10^−5^ at step 2.4 × 10^6^ based on the case shown in Fig. 1c. Note that the chain-breaking rate of DNA (defined as *P*_*BBR*_ × *F*_*BBD*_) is here in effect turned up in an identical scale, which is a conservative assumption considering such a environmental change should be more detrimental to RNA. Nonetheless, the DNA chromosome (red stars) is apparently favored (Fig. 3b, more remarkably at the cellular level, see the lower panel), resulting in a stable platform, which represents a veritable genetic takeover (Additional file 1: Figure S3B). Interestingly and significantly, Fig. 2d shows that such a genetic takeover can originate from a single “individual” harboring the moderate Nrr.

It was suggested that the early RNA world may have occurred in freezing conditions [21, 22] – however, surely such a cold environment could not have last forever. Alternatively, the accomplishment of the genetic takeover may have been driven by the gradual rise of pH value, as described in a hypothesis which proposes that the RNA world originated in an ancient acidic ocean [23]. Even if such an environment change did not happen in the “native habitat”, they could have been associated with “ecological migrations”. For example, as it was described in the “Hot-Cross Origin” hypothesis [24], DNA may have been invented when “RNA creatures” in cool seawater attempted to reach for the hydrothermal vents at seafloor, which harbor more “nutrients” (derived from the thermodynamic disequilibrium therein) but bear an obviously higher temperature. We could envision that in such a scenario, many attempts may have not been successful until once upon a time one mutant carrying a gene of some moderate Nrr joined the migration.

Although the reinforcement of the genetic takeover might have been aided by the alteration of environments, the ultimate irreversibility of the genetic takeover should have arisen accompanying the increase of the genome length, which favored DNA as an internal cause. For example, according the “Hot-Cross Origin” scenario [24], with DNA as their genetic material, the creatures at the hydrothermal vents may have developed to a very complex and advanced extent, so that when they came back to the cooler environment, they eliminated the original RNA inhabitants there. Obviously, for the comeback creatures, an RNA genome would no longer be capable of sustaining their complex living systems.

**Fig. 4:**
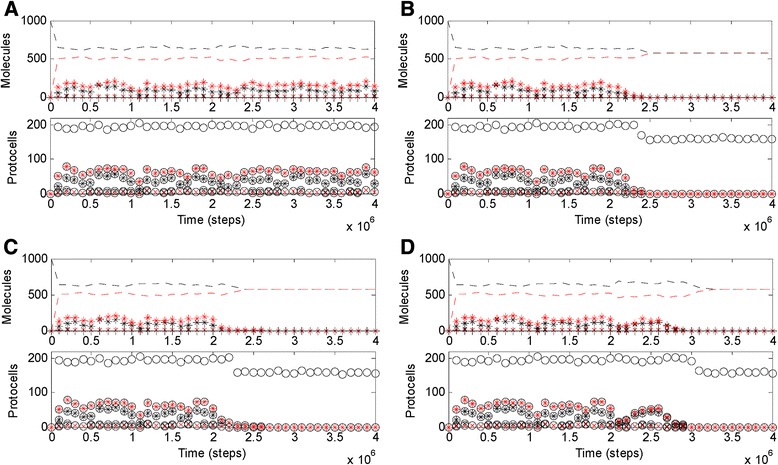
About the causes for the genetic takeover in evolution. Based on the case show in (**a**), which represents an advanced system supported by a DNA genome (See text for details), at step 2 × 10^6^, (**b**) *F*_*SFD*_ is changed from 1 to 2 (the template activity of DNA is turned down); (**c**) *P*_*FPDD*_ is changed from 0.001 to 0.01 (the fidelity of DNA replication is turned down); (**d**) *F*_*BBD*_ is changed from 0.01 to 1 (i.e., DNA becomes equal to RNA in stability)

Corresponding to the ensuing evolution described above, an advanced system depending on DNA was modeled here. The “gene” lengths of Rep, Nsr, Npsr and Arr were changed from 8 to 10 nucleotides (nt), with their corresponding ribozymes’ activities *(P*_*TLR*_, *P*_*NFR*_, *P*_*NPFR*_ and *P*_*AFR*_) enhanced from 0.5 to 0.9. Because Nrr should keep moderate in activity, it was assumed not evolved, its gene remaining 8 nt long and its rate remaining 0.02 (*P*_*NRR*_). With a longer genome, RNA’s template activity should be lower due to its self-folding feature [15], and so *F*_*SFR*_ was changed from 2 to 10. Additionally, total raw material for nucleic acids introduced in the beginning (*T*_*NPPB*_) was increased from 1.8 × 10^5^ to 2 × 10^5^. After the initial inoculation, the protocells contains the DNA chromosome and the ribozymes spread in the system unambiguously (Fig. 4a). When the Nrr’s activity is turned off, with a transient rise of the RNA material (black dashed line) and a simultaneous drop of the DNA material (red dashed line), the system collapses (Additional file 1: Figure S4D), which means that the advanced system is indeed supported by the DNA chromosome and the genetic takeover is no longer reversible. That is a thorough genetic takeover.

### Why genetic takeover – from RNA to DNA?

DNA’s virtues (relative to RNA), i.e., higher stability against hydrolysis [18], better properties as templates [19] and higher fidelity in replication [20], may have accounted for the “dynamic driving force” of the genetic takeover (as mentioned above concerning the benefit aspect of an Nrr). However, a really significant problem is whether these virtues could have contributed to the emergence of larger genomes (which might lead to more complex/efficient life) and thus accounted for the significance of the genetic takeover in evolution.

Based on the DNA-supported advanced system with a larger genome that we have modeled (Fig. 4a), it becomes possible to analyze this problem in a reverse way – by “knocking off” one of the virtues each time. Indeed, when *F*_*SFD*_ is changed from 1 to 2, i.e., the template activity of DNA is turned down to be equal to that of RNA in the primordial system (*F*_*SFR*_ = 2), the system collapses immediately (Fig. 4b); when *P*_*FPDD*_ is changed from 0.001 to 0.01, i.e., the fidelity of DNA replication is turned down to be equal to that of RNA (*P*_*FPRR*_), the system collapses immediately (Fig. 4c); *w*hen *F*_*BBD*_ is changed from 0.01 to 1, i.e., DNA becomes equal to RNA in stability, the system collapses too (Fig. 4d). That is to say, these three distinct virtues are, each in its own way, all important for DNA to sustain an advanced system with a larger genome (see also Additional file 1: Figure S4-left-column for an illustration of the intermediate situation regarding the genome size), so thus they should, all together, have accounted for the significance of the genetic takeover from RNA to DNA in the long-run evolution.

## Discussion

Admittedly, the model used here is complicated (just see the quantity of its parameters, Tables 1 and 2), which attempts to simulate the target system with “micro-resolution” at the level of monomers, such as nucleotides, deoxynucleotides, amphiphiles (membrane components) and their precursors. However, a more abstract model, like the one used in a recent study which addressed the sense of “labor-division” itself to the emergence of DNA – wherein DNA and RNA molecules were treated as impartible “replicators” [25], may be difficult to provide insights as obtained here, especially considering that an Nrr would operate at the level of monomers – catalyzing the reduction of nucleotides. The conclusion draw here should be reliable despite the complexity of the present model: note that actually a large part of the model has already been shown to be reliable or reasonable in a series of previous studies [11–15]; additionally, besides the cases shown in the presented figures, in fact many additional simulations have been run, e.g., with different random seeds, different “key” steps associated with those “turning-down/up” actions and different parameter values, all of which have demonstrated the robustness of the results shown here. On the other hand, to make our model more friendly, in the simulation interface program (Additional file 2), we have provided chance for readers to adjust those parameters that are directly relevant to our study here. The other parameters can also be tested by changing their values in the source code (available on request).

**Fig. 5:**
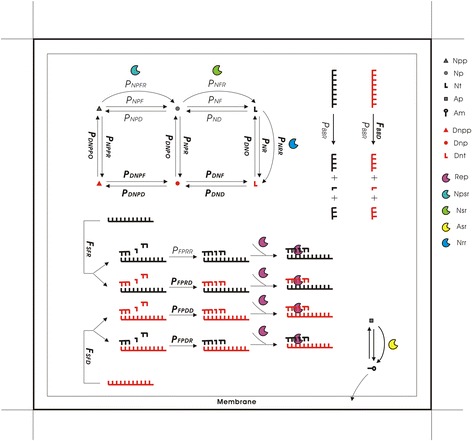
Key events associated with the relation, difference and interaction between RNA and DNA. The diagram shows one grid room in the model which is occupied by a protocell. Legends: Npp, nucleotide precursor’s precursor; Np, nucleotide precursor; Nt, nucleotide; Ap, amphiphile precursor; Am, amphiphile; Dnpp, deoxynucleotide precursor’s precursor; Dnp, deoxynucleotide precursor; Dnt, deoxynucleotide; the notations of ribozymes are the same as in the text. The parameters shown in bold type are those newly introduced which are explained in Table 1, and the others are “old ones” whose descriptions can be found in Table 2. Note that while the rate of chain breaking (hydrolysis) for RNA is represented by *P*_*BBR*_, that for DNA is defined as *P*_*BBR*_ × *F*_*BBD*_ – wherein, *F*_*BBD*_ < <1. The tendency of a nucleic acid chain turning to template is inversely proportional to the factor for its folding, which is *F*_*SFR*_ for RNA and *F*_*SFD*_ for DNA – wherein, *F*_*SFR*_ > *F*_*SFD*_ (and so DNA is easier to act as a template than RNA). The fidelity of the template-directed copying is associated with the probability of false base-pairing tolerated at each residue site when the substrates (monomers or oligomers) are attracted onto the template, which are here represented respectively by *P*_*FPRR*_, *P*_*FPRD*_, *P*_*FPDD*_, and *P*_*FPDR*_ according to the type of the template as well as the type of the substrates. The replication of DNA is more accurate than that of RNA replication – i.e., *P*_*FPDD*_ < *P*_*FPRR*_. See the text for details

In the model, while either DNA or RNA may serve as a template for the copying of the other type of polymer, no DNA/RNA chimeric products (or say, copolymers) may form because it is assumed here that if a foregoing substrate already in place on a template is one type, the subsequent substrates to be incorporated cannot be the other (Fig. 5). On one hand, this is due to the consideration to avoid the model to be further complicated – for the purpose here, this simplification is acceptable because what we intend to focus on is whether one genetic material (DNA) might supersede another (RNA), due to their different properties. On the other hand, there is indeed experimental evidence showing that during the template-depended substrate-addition, the incorporation of a substrate different in type from the foregoing substrate may be much more difficult in reality [26]. Certainly, we cannot rule out the possibility that the chimeric products might form in the process [27] – and they may even have played a role in the genetic takeover considering that such mosaic nucleic acids might appear in some functional forms [28]. Perhaps another modeling study that focuses on this issue is interesting, but still needed is much more knowledge concerning the features of the mosaic nucleic acids, such as how to judge their bond-breaking rate in chain degradation, their fidelity in replication and their folding factor which is related to their tendency to act as a template.

The simulation study here pictures a possible pathway for the genetic takeover, seeded by the emergence of a moderate Nrr, getting spread via the environmental change favoring DNA, and eventually becoming irreversible accompanying the enlargement of the genome. It may be doubted: is the intermediate stage of the environmental change indeed necessary? The answer is likely to be positive. First, if the environment was in favor of DNA (i.e., unfavorable to RNA – in regard of working as the chromosome) at the very beginning, the RNA-based system would have been difficult to arise. For instance, one can enlarge *P*_*BBR*_ in the simulation interface program (Additional file 2) at the start and see the outcome of the simulation. Second, for the genetic takeover from an “established” RNA world, the environmental change should be necessary because the enlargement of the genome should have needed a rather long time in evolution, but the effect of the moderate Nrr is unstable (Fig. 1c) and a locally emerged mutant harboring the Nrr could even hardly sustain itself given that there would always be competitors (those lacking the Nrr) around. The environment change would globalize and stabilize the effect of the Nrr, casting a firm DNA-genome platform for the long-term evolution (Fig. 2d). When the genome became quite large, the environment itself would become unimportant and the genetic takeover was no longer reversible (Additional file 1: Figure S4D).

Although a direct experimental demonstration of an Nrr remains absent, there has already been much evidence showing the chemical plausibility of such a ribozyme [10, 17], especially considering possible aids from some cofactors [29, 30]. The scenario described here may have occurred “quite easily” in the RNA world given that only an Nrr was needed and only a moderate activity was required for the Nrr. Higher activity was even dangerous – the system should seek a tradeoff between the syntheses of the “genomic DNA” and the “functional RNA”. It is actually an important conclusion here that such a systematic balance could be achieved simply by the operation of a single ribozyme with a moderate rate. The kick-off of the genetic takeover by the emergence of such a ribozyme is so simple a way that it ought to have been under the nose of natural selection in the first place. In contrast, an accurate control of DNA and RNA syntheses (e.g., in modern life the syntheses can be separated in different phages in the cell cycle) may have required the existence of quite a few ribozymes; however, apparently it would be difficult to evolve this set of ribozymes simultaneously at the very beginning. Certainly, DNA may have appeared even later – after the invention of proteins [9, 31]. It is here not an aim to argue against this idea; however, considering even a primitive translation system may be quite complicated [10, 17], DNA, if being able to emerge in such a simple way, seems much more likely to have been invented before proteins. Recently, it was seriously considered that due to an alternative, “chemically simpler” way to synthesize deoxynucleotides (i.e., the reversal of deoxyriboaldolase step in deoxynucleotide salvage; perhaps more likely to be within the catalytic capability of RNA), DNA is also possible to have emerged very early [32]. In fact, considering this plausible alternative way, the conclusion of the present modeling study may be stated in a more general tone – that is, in the RNA world, an RNA-to-DNA genetic takeover may simply have been triggered by the emergence of some ribozyme favoring the formation of deoxynucleotides.

As mentioned in the beginning of the paper, significant evolutionary transitions like genetic takeover remains difficult to study experimentally, and they are only “beginning” to appear within the reach of computer simulation researches. Thus it is difficult to find targets to compare concerning other explorations on the topic. One relevant case is an *in silico* simulation study concerning the sense of “labor-division” between template and catalyst to the emergence of DNA in the RNA world [25], as already mentioned above. In that study, it was concluded that DNA being inert in catalytic activity – thus working specially as template, can generate a selective advantage for its emergence in the RNA-based system. The study is not directly comparable to the present study on the aspects concerning mechanisms of the emergence of DNA because neither they address the advantages of DNA as a better template, a more stable polymer and a more faithful information-transmitter, nor we address the issue of labor-division. Indeed, the possible advantage of labor-division is not considered here because before the emergence of DNA, the labor-division is assumed to have already occurred between the ribozymes and the RNA chromosome in our model system [15] – the genetic takeover analyzed here actually takes place from the RNA chromosome to the DNA chromosome. That is, for the scenario of “DNA before proteins”, there is in fact another interesting question: which came first, DNA or the chromosome? Neither of the two studies per se can answer the question.

The result showing that “a certain genetic material, even after it has established its domination, may be substituted by another type of genetic material” can have a more general sense. The replication and expression of genetic information is central to the life phenomenon, and thus it is in fact reasonable to question the plausibility of such a “revolutionary” takeover. That is to say, the present work at the very least, by demonstrating this plausibility, is significant. As a result, indirectly but almost inevitably, it implies that other kinds of genetic takeover were also possible, e.g., the transition from a pre-RNA world to an RNA world [2, 16]. The plausibility of genetic takeover as demonstrated here supports the idea that life, at its core, is a matter of information rather than that of material. That is why the genetic material alters but the living system goes on. Quite interestingly, the pathway of the genetic takeover as pictured here complements that idea with an annotation: in the real world, however, life can only be built upon material, which is constrained by environmental conditions as well as the properties of the material itself. That is why the change of environments might spur the alteration of genetic material, and more significantly, a better genetic material might give rise to a more advanced living system.

## Conclusions

Genetic takeover should be plausible. In the RNA world, such a takeover may simply have been triggered by the emergence of some ribozyme favoring the formation of deoxynucleotides. The transition may initially have been “weak”, but could have been reinforced by environmental changes unfavorable to RNA (such as temperature or pH rise), and would have ultimately become irreversible accompanying the enlargement of genome during the evolution toward complexity. Several distinct virtues of DNA (versus RNA) – higher stability against hydrolysis, greater suitability as template and higher fidelity in replication, should have, each in its own way, all been significant for the genetic takeover in evolution. This simulation study, in a broader sense, enhances our understandings of the relationship between information and material in the living world.

## Methods

The method is in principle the same as those used in our previous studies [11–15], In particular, the present model is derived directly from the one used in a recent study [15]. Therefore, it should be valuable to highlight those new features in the present model, a diagram focusing on the key events that are associated with the relation, difference and interaction between RNA and DNA is provided (Fig. 5).

### A general description

In the model system, each event may occur in a time step (i.e., Monte-Carlo step) with some probability (see Table 1 for the parameters newly introduced and Table 2 for the others). The objects in the model are nucleotide precursors’ precursors, nucleotide precursors, nucleotides (A, G, C, and U), RNA, deoxynucleotide precursors’ precursors, deoxynucleotide precursors, deoxynucleotides (dA, dG, dC and dU; dT is not considered and is assumed to appear later in evolution), DNA, amphiphile precursors, amphiphiles, and protocells. An *N* × *N* grid is used for the system, with toroidal topology to avoid edge effects. Molecules may move from one “grid room” to an adjacent one, but within one step, only molecules within the same grid room can interact. Membrane may assemble (from amphiphiles) at the edge of a grid room and then the grid room is occupied by a protocell. When a protocell moves to an adjacent naked grid room, the protocell would push away molecules in that room. When a protocell divides, amphiphiles on the membrane and molecules in the protocells would be distributed randomly between the two offspring protocells; one of the offspring protocells would occupy an adjacent naked grid room and push away molecules in that room. Only protocells at adjacent grid rooms can fuse to each other.

There are background (non-enzymatic) inter-conversions between deoxynucleotides and nucleotides, between deoxynucleotide precursors and nucleotide precursors, and between deoxynucleotide precursors’ precursors and nucleotide precursors’ precursors – as a case supporting such assumptions, it has been demonstrated in a recent experimental study that nucleotide precursors might transform into deoxynucleotide precursors abiotically [33].

DNA molecules would not function as catalysts, whereas an RNA molecule, if in the form of single (linear) strand and containing a characteristic sequence domain (presumed arbitrarily, here only 8–10 nt long for simplification) may function as a corresponding ribozyme: Rep, Npsr, Nsr, Asr or Nrr. However, the catalytic RNA should be shorter than 1.5 times of the characteristic domain; otherwise, it is deemed that the “correct” structure would be interfered by the redundant residues and it would not act as the ribozyme. Here Rep is assumed to be able to aid all the four types of copying: RNA to RNA, RNA to DNA, DNA to DNA and DNA to RNA. Note that the type of the product, RNA or DNA, is assumed to depend on the foregoing substrate – if a foregoing substrate on a template is one type, the subsequent substrates to be incorporated cannot be the other type (thus, no DNA/RNA chimeric product, or say “copolymer”, would form; see discussion for an explanation on this assumption) (Fig. 5). Nrr, the key ribozyme newly introduced here, can catalyze the transformation of nucleotides to deoxynucleotides.

**Fig. 6:**
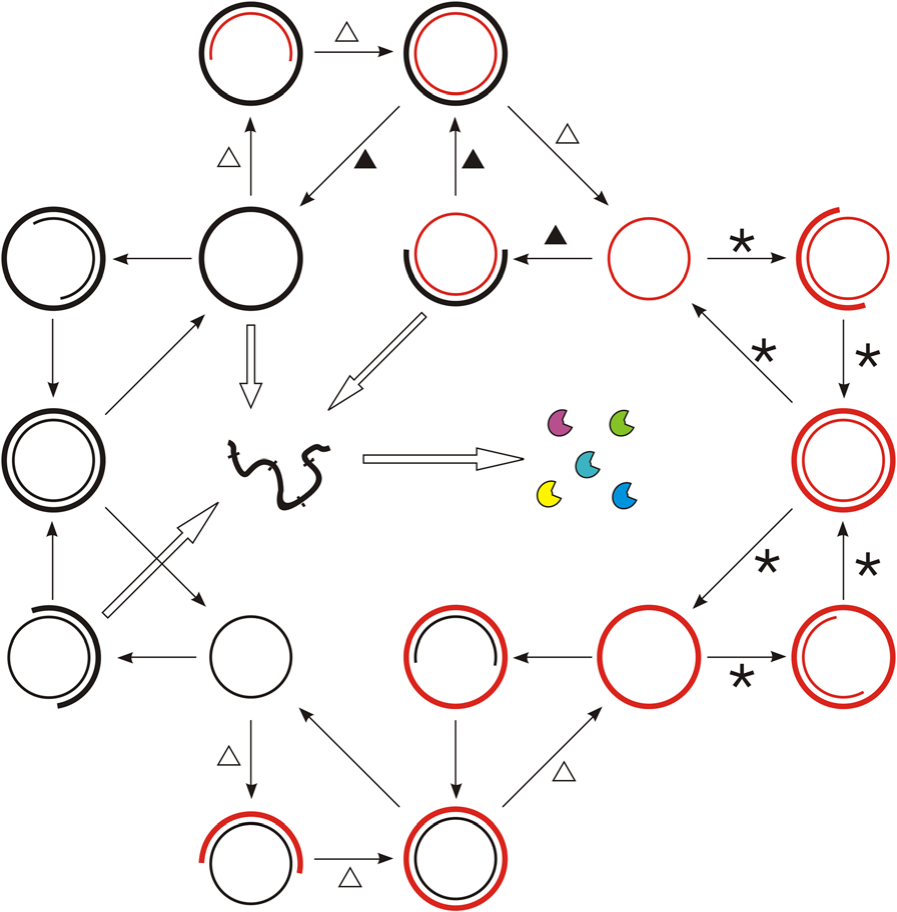
A scheme describing the routes concerning replication and transcription in the system. The chromosome is in a circular form: thick lines represent the sense chain, while thin lines represent the antisense chain. RNA is drawn in black, while DNA in red. Broad white arrows highlight the way in which the ribozymes (*crescent-shapes*) are created: the linear sense RNA, which arises from the spontaneous break of the sense chain of the RNA chromosome or partial copying of the antisense chain of the RNA or DNA chromosome, may be readily broken at sites (*marked by short bands*) between the linked genes (the so-called “self-cleaving” feature, see [15] for details). The routes which are led in the original pure RNA-based system can be easily identified at the left part, where no DNA (*red line*) is involved. When Nrr begins to take effect, “reverse transcription” may happen and give rise to the DNA chromosome (*see the routes labeled by white triangles*). When a thorough genetic takeover is completed, DNA’s replication (*labeled by stars*) and its transcription to the sense RNA chain (*labeled by black triangles*) would constitute the most significant information flow in the living system

The RNA chromosome (sense chain) has a sequence of the ribozyme domains (i.e., genes) in a tandem and circular way [15]. Note that the RNA chromosome would not be able to act as a ribozyme, because it is in a circular form (in the model, only linear RNA could fold into ribozyme) – in fact, here, even it is in a linear form, it would not act as a ribozyme, because the chromosome, comprising five ribozyme domains, would be much longer than 1.5 times of one ribozyme domain. The DNA chromosome, which origins from the “reverse transcription” of the RNA chromosome, has an identical sequence with the RNA chromosome. The routes concerning the replication and inter-copying of the RNA chromosome and the DNA chromosome, as well as the creation of the ribozymes, are summarized in a sketch (Fig. 6).

Though it has been demonstrated that RNA of only 5–7 nt long may work as ribozymes (reviewed in ref [3]), it seems unlikely that so short (or a little longer, such as 8-10 nt adopted here) an RNA molecule can work as any of the ribozymes assumed in the present model. As a consequence, such a genome with a size of only 40–50 nt is also quite unrealistic. In fact, as mentioned above, the type of our model has a “micro-resolution” – events occurred in the system are described at the level of monomers (deoxy/nucleotides for DNA/RNA and single amphiphilic molecules for membrane), and the computation is very intensive for so complicated a model system like the present one. The main purpose for that we set the presumed ribozyme-characteristic sequences no longer than 10 nt is to avoid cumbersome computation, based on the understanding that the key relation, i.e., the sequence-function correspondence of these ribozymes, is maintained in the simulation.

In a simulation case, a quantity of nucleotide precursors’ precursors and amphiphile precursors are introduced initially, some empty protocells are inoculated soon after the initial step, and some protocells containing a few molecules of the ribozymes and the chromosome (RNA or DNA, according to the purpose of the simulation case) are inoculated some steps later. These interventions, taken together, are called “the initial inoculation” here. Then, only “internal” events in the model (see below for details) govern the whole dynamic process, except that at some “key” step, a certain parameter may be turned down or up to show its influence.

Here, to provide readers an intuition to our simulation, we have constructed a simulation interface program (Additional file 2). The program demonstrates the case shown in Fig. 3b as an example, but one can run the program in his own way by adjusting parameters (those directly relevant to the present study). Notably, the parameters can be adjusted in real time, so the turning-down/up actions, which are adopted frequently throughout our study here, can be experienced freely.

### Events occurring in a time step and associated parameters

A nucleotide precursor’s precursor may form a nucleotide precursor, with the probability *P*_*NPF*_ (see Tables 1 and 2 for a description of this abbreviation and those appearing below) in a non-enzymatic way, or with *P*_*NPFR*_ when catalyzed by an Npsr. A deoxynucleotide precursor’s precursor may form a deoxynucleotide precursor, with the probability *P*_*DNPF*_ (non-enzymatic). A nucleotide precursor may form a nucleotide (randomly as A, U, C, or G) with *P*_*NF*_ in a non-enzymatic way, or with *P*_*NFR*_ when catalyzed by an Nsr. A deoxynucleotide precursor may form a deoxynucleotide (randomly as dA, dU, dC, or dG) with *P*_*DNF*_ (non-enzymatic). A nucleotide may decay into a nucleotide precursor with *P*_*ND*_. A deoxynucleotide may decay into a deoxynucleotide precursor with *P*_*DND*_. A nucleotide residue at the end of an RNA chain may decay into a nucleotide precursor with *P*_*NDE*_, A deoxynucleotide residue at the end of a DNA chain may decay into a deoxynucleotide precursor with *P*_*DNDE*_, A nucleotide precursor may decay into a nucleotide precursor’s precursor with *P*_*NPD*_. A deoxynucleotide precursor may decay into a deoxynucleotide precursor’s precursor with *P*_*DNPD*_. A nucleotide may transform to a deoxynucleotide with *P*_*NR*_ in a non-enzymatic way, or with *P*_*NRR*_ when catalyzed by an Nrr. A deoxynucleotide may transform to a nucleotide with *P*_*DNO*_ (non-enzymatic). A nucleotide precursor may transform to a deoxynucleotide precursor with *P*_*NPR*_ (non-enzymatic). A deoxynucleotide precursor may transform to a nucleotide precursor with *P*_*DNPO*_ (non-enzymatic). A nucleotide precursor’s precursor may transform to a deoxynucleotide precursor’s precursor with *P*_*NPPR*_ (non-enzymatic). A deoxynucleotide precursor’s precursor may transform to a nucleotide precursor’s precursor with *P*_*DNPPO*_ (non-enzymatic). See Fig. 5 for a more intuitional description of these inter-transforming reactions and relevant probabilities.

Free of template, two RNA (or DNA) molecules may be ligated with *P*_*RL*_, forming longer RNA (or DNA) chains. An RNA (or DNA) molecule may be ligated end-to-end with *P*_*EL*_, forming a circular chain. A phosphodiester bond within an RNA chain may break with *P*_*BBR*_. A phosphodiester bond within an DNA chain may break with *P*_*BBR*_ × *F*_*BBD*_ (wherein, *F*_*BBD*_ < <1, see the last section for the explanations concerning the setting of parameters). For a particular intermediate site between two genes in a linear RNA, *P*_*BBR*_ is multiplied by a factor, *F*_*IB*_ (>1), which represents the consideration of the self-cleaving effect [15].

A linear RNA may turn into a template (unfolding) with *P*_*LTT*_ / F_SFR_, whereas a circular RNA may turn into a template with *P*_*CTT*_ / *F*_*SFR*_. A linear DNA may turn into a template (unfolding) with *P*_*LTT*_ / *F*_*SFD*_, whereas a circular DNA may turn into a template with *P*_*CTT*_ / *F*_*SFD*_. A template may attract monomers or oligomers as substrates with *P*_*AT*_ by base-pairing. When a template attracts a substrate, if the template is RNA and the substrate is also of RNA (i.e., a nucleotide or a oligo-nucleotide), the probability of false base-pairing tolerated at each residue site is *P*_*FPRR*_; when the template is RNA and the substrates are of DNA (i.e., deoxynucleotides or oligo-deoxynucleotides), the false base-pairing probability is *P*_*FPRD*_; when the template is DNA and the substrates are also of DNA, the false base-pairing probability is *P*_*FPDD*_; when the template is DNA and the substrates are of RNA, the false base-pairing probability is *P*_*FPDR*_ (Fig. 5).

Monomers and oligomers aligned adjacently on a template may be ligated to each other with *P*_*TL*_ (template-directed ligation) in a non-enzymatic way. A Rep molecule may bind onto a template with *P*_*RB*_ and drop from the template with *P*_*RD*_. When there is a Rep on the template, the template-directed ligation may occur with *P*_*TLR*_; however, if one or both base pairs flanking the ligation site are false, the ribozymatic ligation would not occur unless another probability, *P*_*FLR*_, is satisfied. The substrates or the final product (the complementary chain) may dissociate from the template if base pairs between them can separate (each base pair may separate with *P*_*SP*_).

An amphiphile precursor may transform to an amphiphile with *P*_*AF*_ in a non-enzymatic way, or with *P*_*AFR*_ when catalyzed by an Asr. Amphiphiles (with a lower limit of quantity *L*_*AM*_) may assemble into a membrane at the edge of a grid room with *P*_*MF*_, encompassing molecules within it and forming a “protocell”. A free amphiphile may decay into an amphiphile precursor with *P*_*AD*_, whereas an amphiphile within a protocell membrane (not within the protocell but only on the membrane) may decay with *P*_*ADM*_. A free amphiphile may join the membrane of a protocell with *P*_*AJM*_*,* whereas an amphiphile within a protocell membrane may leave it with *P*_*ALM*_. Nucleotides, deoxynucleotides, RNA and DNA are assumed to be impermeable, whereas a nucleotide precursor’s precursor or a deoxynucleotide precursor’s precursor may diffuse across the membrane with *P*_*NPPP*_; a nucleotide precursor or a deoxynucleotide precursor may diffuse with *P*_*NPP*_; and an amphiphile precursor may diffuse with *P*_*APP*_. A protocell may divide into two with *P*_*CD*_ and two adjacent protocells may fuse into one with *P*_*CF*_. A protocell may break (its membrane disassembling into free amphiphiles) with *P*_*CB*_. The probabilities of the decay of a nucleotide precursor (*P*_*NPD*_), a nucleotide (*P*_*ND*_), a nucleotide residue at the end of an RNA chain (*P*_*NDE*_), a deoxynucleotide precursor (*P*_*DNPD*_), a deoxynucleotide (*P*_*DND*_), a deoxynucleotide residue at the end of a DNA chain (*P*_*DNDE*_), and the probability of phosphodiester bond breaking (*P*_*BBR*_ for RNA and *P*_*BBR*_ × *F*_*BBD*_ for DNA), are multiplied by a factor, *F*_*DO*_ (>1), when these events occur out of protocells. This represents the consideration that water activity should be higher outside the protocells.

Before the next time step, molecules and protocells in a grid room may move into adjacent rooms. A nucleotide, a nucleotide precursor, a nucleotide precursor’s precursor, a deoxynucleotide, a deoxynucleotide precursor, a deoxynucleotide precursor’s precursor, an amphiphile, or an amphiphile precursor may move with *P*_*MV*_, whereas a protocell may move with *P*_*MC*_.

### Some detailed assumptions considering real situations

The probability of the separation of an RNA (or DNA) template with its substrates or the final product (the complementary chain) is actually assumed to be *P*_*SP*_^*q*^, wherein *q* equals to *r*^1/2^, and *r* is the number of base pairs in the duplex. When *r* increases and thus *q* increases, *P*_*SP*_^*q*^ would decrease (because *P*_*SP*,_ as a probability, has a value between 0 and 1). That is, the separation of the two strands would be more difficult if the base pairs are more. The introduction of the square root (i.e., *q* equals to *r*^1/2^) represents the consideration of the synergistic effect of the separation of the base pairs (if a base pair is separated, the base pairs flanking it would be easier to separate).

The probability of membrane formation is assumed to be 1-(1-*P*_*MF*_)^x^, where x equals to *a*-*L*_*AM*_ + 1, and *a* is the number of amphiphiles in the grid room. When *a* equals to *L*_*AM*_, the probability of membrane formation equals to *P*_*MF*_. This assumption concerns the consideration that the more amphiphiles there are, the more possible it is that they would assemble to form a cell membrane.

The probability of an amphiphile leaving the membrane is assumed to be *P*_*ALM*_ / [1 + *F*_*OP*_ × *n*/(*b*/2)^3/2^], where *n* is the quantity of inner impermeable ions, including nucleotides/deoxynucleotides and RNA/DNA (measured by the number of nucleotide/deoxynucleotide residues), and *b* is the quantity of amphiphiles within the membrane. Wherein, *b*/2 (there are two layers in the membrane) is a “scale” representation of the surface area of the membrane. Consequently, (*b*/2)^3/2^ is a scale representation of the cellular space. Thus, *n*/(*b*/2)^3/2^ is a representation of the concentration of the ions. *F*_*OP*_ × *n*/(*b*/2)^3/2^ represents the consideration for the “osmotic pressure effect”; a higher concentration of the inner impermeable ions would cause the protocell to be more swollen, and thus amphiphiles within the membrane are less likely to leave [14].

The probability of a nucleotide (or deoxynucleotide) precursor permeating into a protocell is assumed to be [1-(1-*P*_*NPP*_)^y^] / [1 + *F*_*DE*_ × *n*/(*b*/2)^3/2^], where *n* is the quantity of inner impermeable ions and *b* is the quantity of amphiphiles within the membrane. The index y equals to (*b*/*L*_*AM*_)^3/2^, which represents the consideration of the limiting effect of the cellular space on the influx of nucleotide (or deoxynucleotide) precursors. When *b* equals to *L*_*AM*_ (the lower limit of the number of amphiphiles to form a protocell membrane), y equals to 1. When the *b* increases, meaning that the cellular space increases correspondingly, the probability of a nucleotide (or deoxynucleotide) precursor permeating into the protocell would become greater. *F*_*DE*_ × *n*/(*b*/2)^3/2^ represents the consideration of the effect of Donnan’s equilibrium – see [13] for a detailed explanation of the influence of Donnan’s equilibrium on the protocell. Similarly, the probability of a nucleotide (or deoxynucleotide) precursor’s precursor permeating into a protocell is assumed to be [1-(1-*P*_*NPPP*_)^y^] / [1 + *F*_*DE*_ × *n*/(*b*/2)^3/2^]. However, the probability of an amphiphile precursor permeating into a protocell is assumed to be 1-(1-*P*_*APP*_)^y^, wherein the effect of Donnan’s equilibrium is not considered because amphiphile precursors are assumed to be uncharged molecules.

The probability of protocell division is assumed to be *P*_*CD*_ × (1-2 × *L*_*AM*_/*b*), where *b* is the quantity of amphiphiles within the membrane. When *b* is no more than twice that of *L*_*AM*_ (the lower limit of the number of amphiphiles to form a protocell membrane), the probability is no more than 0, i.e., the protocell could not divide. This assumption represents the consideration that the larger the protocell, the more the probability that it would divide.

The probability of the movement of an RNA (or DNA) molecule is assumed to be *P*_*MV*_/ *m*^1/2^, where *m* is the polymer’s mass relative to a monomer (i.e., the residue number). This assumption represents the consideration of the effect of the molecular size on the molecular movement. The square root was adopted here according to the prediction of the Zimm model concerning the diffusion coefficient of polymer molecules in solution [34], in agreement with corresponding experiments. Note that this formula is somewhat different from the one used in our previous models, in which *m*^1/3^ was used. We have not found any apparent difference concerning our results that this change may bring about, but now that we have relevant data coming from experiments, we would like to modify our model according to such knowledge.

### Logical setting of the parameters in the model

Notably, there are several key points in the parameter setting of the present model, which are associated with the topic of genetic takeover. First, because DNA is much more stable against hydrolysis than RNA [18], *F*_*BBD*_ should be significantly smaller than 1, Second, because DNA is less likely to self-fold (and thus acts better as a template [19]), *F*_*SFD*_ should be apparently smaller than *F*_*SFR*_. Third, according to a recent study which showed that the “intrinsic” fidelities of the different kinds of template-directed copying between DNA and RNA are different from each other [20], DNA replication has the highest fidelity – thus *P*_*FPDD*_ should be the lowest among the four kinds of false base-pairing probabilities that are assumed in this model; DNA-directed RNA synthesis has the lowest fidelity – thus *P*_*FPDR*_ should be the highest; RNA-directed DNA synthesis and RNA replication are similar in fidelity – thus *P*_*FPRD*_ ≈ *P*_*FPRR*_.

Certainly, there should be quite a few other constrains for an appropriate parameter setting. Ribozymatic reactions should be much more efficient than corresponding non-enzymatic reactions, so *P*_*TLR*_> > *P*_*TL*_, *P*_*NPFR*_> > *P*_*NPF*_, *P*_*NFR*_> > *P*_*NF*_, *P*_*NRR*_> > *P*_*NR*_, and *P*_*AFR*_> > *P*_*AF*_. Those non-enzymatic “inter-transforming” rates between RNA’s and DNA’s precursors, i.e., *P*_*NR*_, *P*_*DNO*_, *P*_*NPR*_, *P*_*DNPO*_, *P*_*NPPR*_, and *P*_*DNPPO*_, should be quite low and of the same order. “Template-directed ligation” should be significantly more efficient than “random ligation”, so *P*_*TL*_> > *P*_*RL*_. The end-to-end ligation of an RNA (or DNA) chain (i.e., cyclization) should be similar in efficiency to the random ligation of two different RNA (or DNA) chains, so *P*_*EL*_ should be of the same order as *P*_*RL*_. Nucleotide (or deoxynucleotide) residues in an RNA (or DNA) chain should be protected. And so here, nucleotide (or deoxynucleotide) residues within the chain are assumed to be unable to decay, whereas a nucleotide (or deoxynucleotide) residue at the end of a chain, which is only “half protected”, may decay, but at a rate lower than that of free nucleotides (or deoxynucleotides), i.e., *P*_*NDE*_ < *P*_*ND*_ and *P*_*DNDE*_ < *P*_*DND*_. Amphiphiles within a membrane should be protected, so *P*_*ADM*_ < *P*_*AD*_. Because of the self-assembly feature of amphiphilic molecules, *P*_*MF*_> > *P*_*CB*_ and *P*_*AJM*_> > *P*_*ALM*_. The movement of molecules should be easier than protocells, so *P*_*MV*_ > *P*_*MC*_. A nucleotide (or deoxynucleotide) precursor’s precursor should be easier to permeate through the membrane than a nucleotide (or deoxynucleotide) precursor, so *P*_*NPPP*_ > *P*_*NPP*_. Other considerations may include: *P*_*BB*_ and *P*_*RL*_ may be of the same order, *P*_*ND*_ ≥ *P*_*NF*_, *P*_*NPD*_ ≥ *P*_*NPF*_, *P*_*DND*_ ≥ *P*_*DNF*_, *P*_*DNPD*_ ≥ *P*_*DNPF*_, *P*_*AD*_ ≥ *P*_*AF*_, *P*_*APP*_ ≥ *P*_*NPP*_, and so on. To set parameters “appropriately”, according to logic and our knowledge, represents an effort to make the “behavior” of our model system relevant to that of its target system in reality.

(Note: The source code of the simulation program, written in C language, can be obtained from the corresponding author on request by e-mail. The source code could help readers to understand the model better if they have sufficient background knowledge in programming.)

## Availability of supporting data

All the supporting data are included as additional files.

## Additional files

Additional file 1: Figs-S1-S4. This file includes four supporting figures for the paper, Figs. S1-S4, which have been cited correspondingly in the text. (PDF 471 kb) Additional file 2:
**gt1-dx.** This file is an executable version of our simulation interface program, which has been tested under the platform of MS-Windows XP and MS-Windows 7.0. (EXE 3434 kb)

## Abbreviations

Asr: The amphiphile synthetase ribozyme
Nrr: The nucleotide reductase ribozyme
Nsr: The nucleotide synthetase ribozyme
Nspr: The nucleotide precursor synthetase ribozyme
nt: Nucleotide
Rep: The replicase ribozyme
