# Supplementary material for: The emergence of DNA in the RNA world: an in silico simulation study of genetic takeover
Source: BMC Evol Biol. 2015 Dec 7;15:272. doi: 10.1186/s12862-015-0548-1 (PMC4672488; doi:10.1186/s12862-015-0548-1)
Supplement: Additional file 1: Figs-S1-S4. — This file includes four supporting figures for the paper, Figs. S1-S4, which have been cited correspondingly in the text. (PDF 471 kb) [file 12862_2015_548_MOESM1_ESM.pdf]

Four supporting figures (Figs. S1-S4) for

"The emergence of DNA in the RNA world: an *in silico* simulation study of genetic takeover"

Wentao Ma*, Chunwu Yu, Wentao Zhang, Sanmao Wu and Yu Feng

* Email: mwt@whu.edu.cn

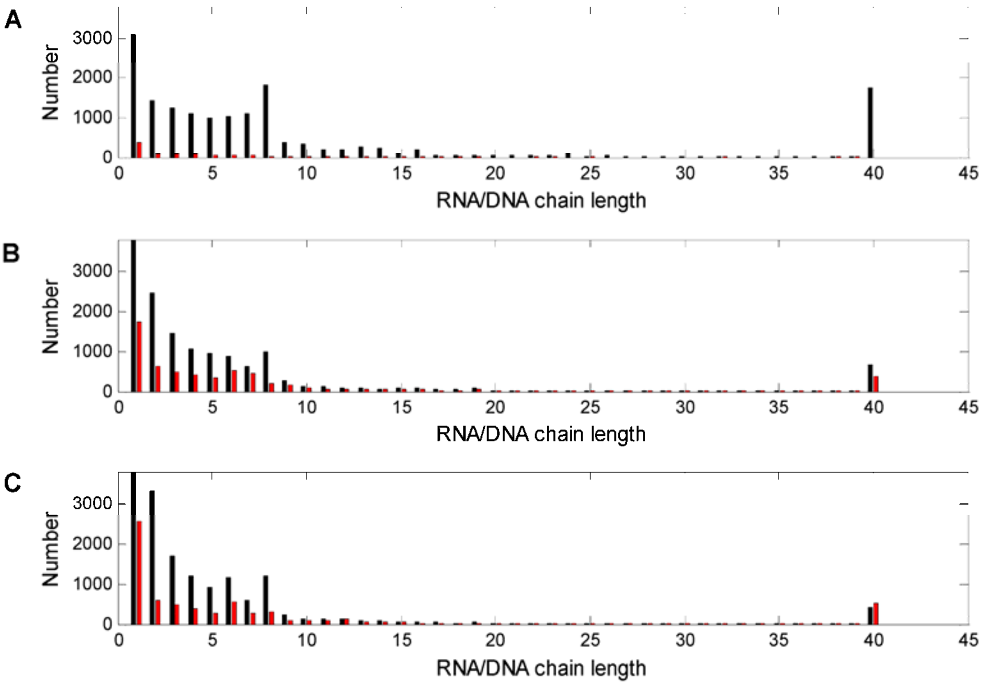

Fig. S1. The chain length distribution of nucleic acids at certain steps in some simulation cases. (**A**) At step 3×10^6 in the case shown in Fig. 1A. (**B**) At step 1.5×10^6 in the case shown in Fig. 1C. (**C**) At step 3×10^6 in the case shown in Fig. 3A. Black bars denote RNA while red bars denote DNA. The number of mononucleotides is not fully presented in *B* (actually 7,227) and *C* (actually 11,382). Note that in these cases the length of the chromosome is 40 nt, including five 8 nt genes – i.e., the genes for Rep, Nsr, Npsr, Asr and Nrr.

.

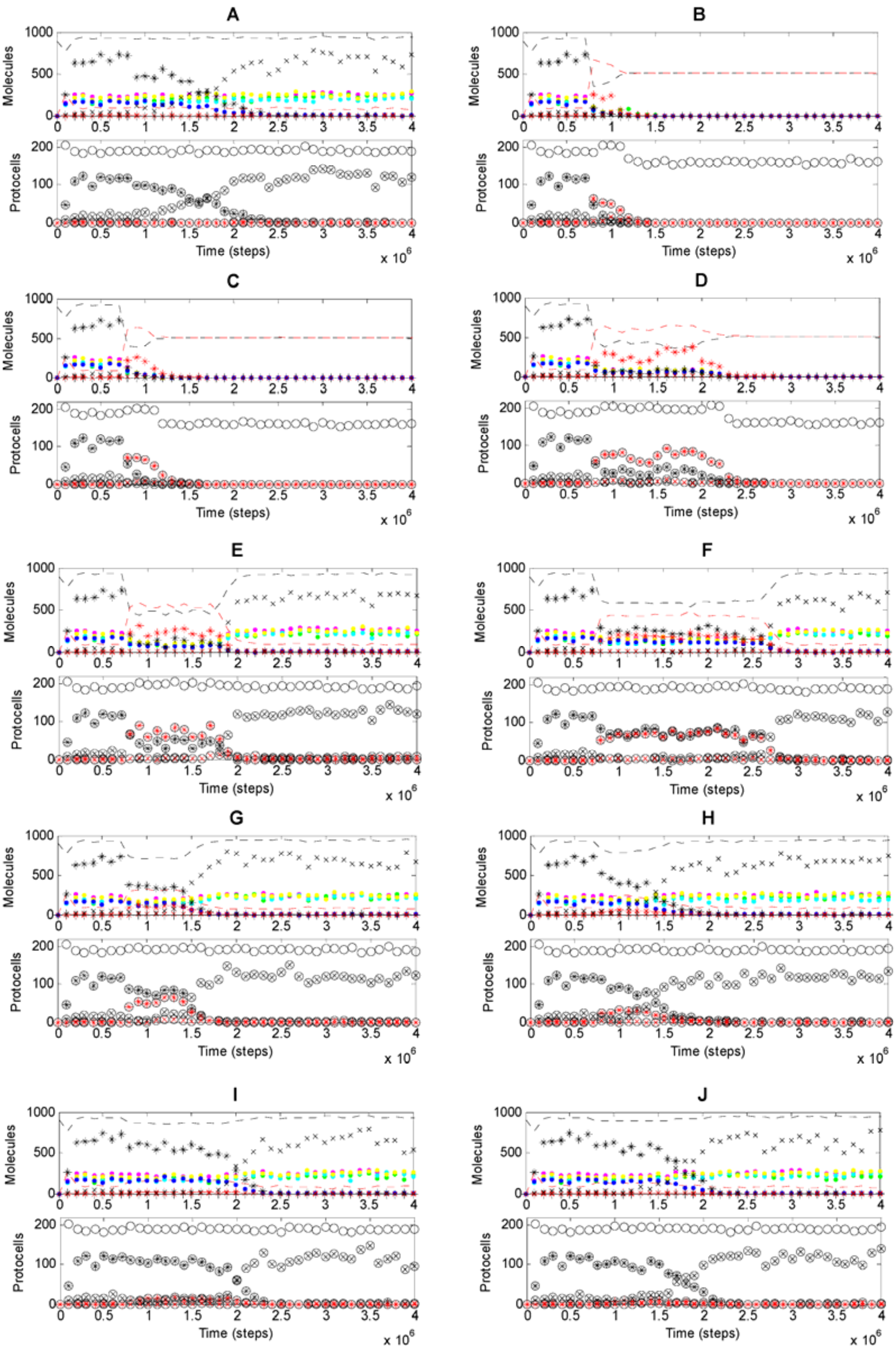

Fig. S2. The influence of Nrr with different active rates on the system of the RNA-based protocells (an extended version)., Based on the case shown in (**A**), at step 0.7×10^6, P_{NRR} is changed from 0 to (**B**) 0.5; (**C**) 0.2; (**D**) 0.1; (**E**) 0.05; (**F**) 0.02; (**G**) 0.01; (**H**) 0.005; (**I**) 0.002; and (**J**) 0.001. Note that here *A*, *B*, *F* and *J* are identical to Fig. 1A, B, C and D respectively. With regard to the length of the DNA/RNA platform, 0.02 appears to be the most appropriate rate for the Nrr here.

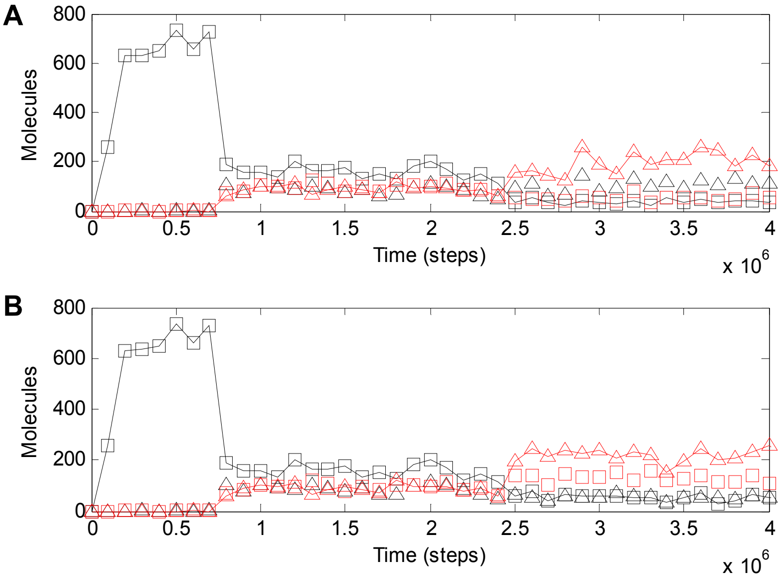

Fig. S3. The stabilization of the Nrr-induced DNA/RNA platform engenders a veritable genetic takeover. (**A**) corresponds to the case shown in Fig. 3A, and (**B**) corresponds to the case shown in Fig. 3B. The RNA chromosome is drawn in black, whereas the DNA chromosome in red. Squares denote the chromosome descending from RNA (i.e., using an RNA chromosome as the template in its synthesis), whereas triangles denote the chromosome descending from DNA. In fact, the numbers represented by a black square and a black triangle at a time step, if adding up, would equal to the number represented by a black star at the corresponding step in Fig. 3 (the total number of the RNA chromosome); similarly, a red square plus a red triangle would make a corresponding red star in Fig. 3 (the total number of the DNA chromosome). Black squares (i.e., the RNA chromosome coming from their own replication) and red triangles (i.e., the DNA chromosome coming from their own replication) are specially threaded with lines to highlight their significance as a sign of the genetic takeover – that is, ultimately, the DNA chromosome not only dominates the system but also can sustain itself by its own replication.

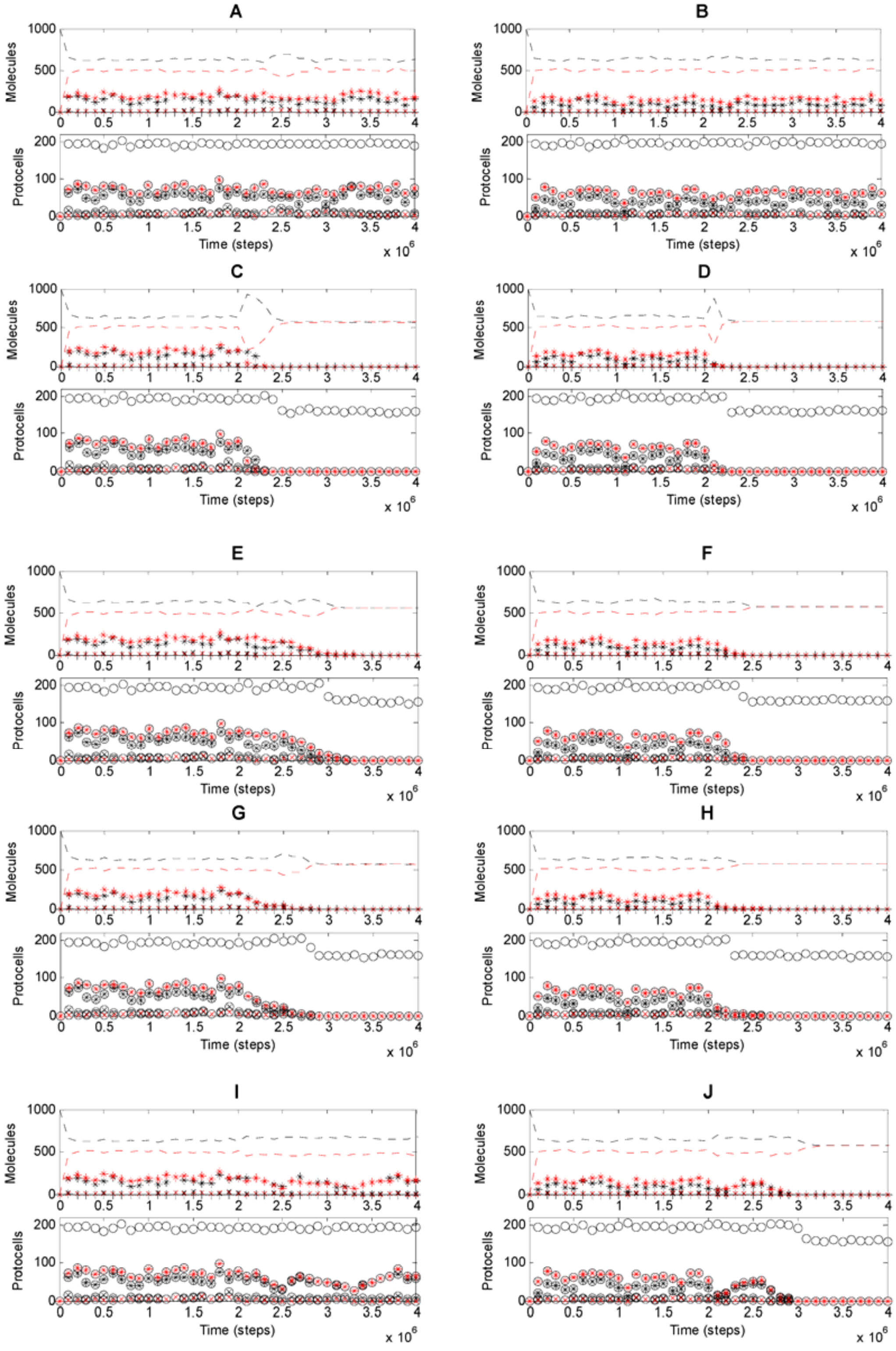

Fig. S4. About the causes for the genetic takeover in evolution (an extended version). Here *B*, *F*, *H* and *J* are identical to Fig. 4A, B, C and D respectively. Based on the case shown in (**B**), which represents the spread of a DNA-dependent system with a genome length of 48 nt (each ribozyme domain 10 nt long except for the Nrr – 8 nt), at step 2×10^6, (**D**) P_{NRR} is changed from 0.02 to 0; (**F**) F_{SFD} is changed from 1 to 2; (**H**) P_{FPDD} is changed from 0.001 to 0.01; (**J**) F_{BBD} is changed from 0.01 to 1. The corresponding cases in the left column, i.e., (**A**), (**C**), (**E**), (**G**) and (**I**), are

interpreted the same way except that they are associated with a 44-nt DNA genome (each ribozyme domain 9 nt long except for the Nrr – 8nt). The left column represents an intermediate stage before the development of the evolved chromosome as shown in the right column. The results reflect the transitional situation well. When the template activity or the fidelity of DNA is turned down halfway, the "intermediate" system also collapses but apparently with a gentler tendency (*E*, *G*) compared with the corresponding cases for the evolved system (*F*, *H*). When the stability of DNA is turned down, the intermediate system is "shaken" and becomes "dangerous" (*I*); however, it does not collapse (at least not before the final step shown here), unlike the corresponding case for the evolved system (*J*).
